# Antineoplastic and Apoptotic Potential of Traditional Medicines Thymoquinone and Diosgenin in Squamous Cell Carcinoma

**DOI:** 10.1371/journal.pone.0046641

**Published:** 2012-10-15

**Authors:** Subhasis Das, Kaushik Kumar Dey, Goutam Dey, Ipsita Pal, Abhijit Majumder, Sujata MaitiChoudhury, Subhas C. kundu, Mahitosh Mandal

**Affiliations:** 1 School of Medical Science and Technology, Indian Institute of Technology-Kharagpur, West Bengal, India; 2 Department of Clinical Cancer Prevention, The University of Texas MD Anderson Cancer Center, Houston, Texas, United States of America; 3 Department of Human Physiology with Community Health, Vidyasagar University, West Midnapur, West Bengal, India; 4 Department of Biotechnology, Indian Institute of Technology-Kharagpur, West Bengal, India; Massachusetts Eye & Ear Infirmary, Harvard Medical School, United States of America

## Abstract

Thymoquinone (TQ) and diosgenin (DG), the active ingredients obtained from black cumin (*Nigella sativa*) and fenugreek (*Trigonella foenum graecum*), respectively, exert potent bioactivity, including anticancer effects. This study investigated the antineoplastic activity of these agents against squamous cell carcinoma *in vitro* and sarcoma 180–induced tumors *in vivo*. TQ and DG inhibited cell proliferation and induced cytotoxicity in A431 and Hep2 cells. These agents induced apoptosis by increasing the sub-G_1_ population, LIVE/DEAD cytotoxicity, chromatin condensation, DNA laddering and TUNEL-positive cells significantly (*P*<0.05). Increased Bax/Bcl-2 ratio, activation of caspases and cleavage of poly ADP ribose polymerase were observed in treated cells. These drugs inhibited Akt and JNK phosphorylations, thus inhibiting cell proliferation while inducing apoptosis. In combination, TQ and DG had synergistic effects, resulting in cell viability as low as 10%. In a mouse xenograft model, a combination of TQ and DG significantly (*P*<0.05) reduced tumor volume, mass and increased apoptosis. TQ and DG, alone and in combination, inhibit cell proliferation and induce apoptosis in squamous cell carcinoma. The combination of TQ and DG is a potential antineoplastic therapy in this common skin cancer.

## Introduction

Skin cancer is the most common form of human cancer. It is estimated that over one million new cases occur annually. The annual rates of all forms of skin cancer are increasing each year worldwide. The two most common forms of skin cancer are basal cell carcinoma and squamous cell carcinoma (SCC). Together, these two are referred to as nonmelanoma skin cancer. SCC is the second most common form of nonmelanoma skin cancer and accounts for 20% of cutaneous malignancies [1], [2].

In recent years, the use of herbal therapies for the prevention and treatment of cancer is increased. Thymoquinone (TQ), one of the active ingredients of the volatile oil of black cumin (*Nigella sativa* L.), is used for medicinal purposes for more than 2000 years. The black seed herb grows in India and countries bordering the Mediterranean Sea and is used in the Middle East since long and Africa to promote health and fight diseases [3]. Thymoquinone is reported to exhibit antioxidant, anti-inflammatory and anticancer activities [3], [4]. While the cytotoxicity of this compound is reported in many different types of malignancy; including breast cancer, prostate cancer, osteosarcoma, myeloblastic leukemia and colorectal carcinoma [5]–[7]. It has minimal toxicity in normal cells [8]. It has exhibited promising antitumor activity in murine tumor models xenografted with colon cancer [3] or prostate cancer [9]. Thymoquinone is shown to induce antitumor effects mediated via peroxisome proliferator-activated receptor gamma, p53-dependent and p53-independent pathways. It upregulates p53 and p21 in HCT116 cells resulting in inhibition of antiapoptotic Bcl-2 protein [6]. It inhibits the proliferation of a panel of human colon cancer cells (Caco-2, HCT-116, LoVo, DLD-1 and HT-29) by increasing the phosphorylation states of the mitogen-activated protein kinases (MAPK) JNK and ERK, but not of p38 [10].

Diosgenin (DG), a bioactive component found in fenugreek (*Trigonella foenum graecum*) and the roots of the wild yam (*Dioscorea villosa*), is a traditional medicine. It is used in the treatment of hypercholesterolemia, hypertriacylglycerolemia, and diabetes and hyperglycemia [11]. Published reports reveal that DG inhibits proliferation and induces apoptosis in a wide variety of human tumor cells: colon, breast, prostate and liver as well as osteosarcoma and leukemia [12]–[13]. The antiproliferative and apoptotic properties of DG are suggested by its ability to arrest the cell cycle, activate p53, release apoptosis-inducing factor and modulate caspase-3 activity [14]. Other reports suggest that DG inhibits the ERK, JNK and phosphoinositide 3-kinase (PI3K)/Akt signaling pathways, nuclear factor kappa B (NF-κB) activity and NF-κB–regulated gene expression, subsequently reducing proliferation, migration and invasion by reducing matrix metalloproteinases and osteoclastogenesis [12], [15].

Although the anticancer and proapoptotic properties of TQ and DG are reported in many studies, their effects in SCC, alone or in combination are not fully explored. Combining the two highly bioactive agents offers a powerful approach to cancer treatment and may counteract such a way, that the efficacy of antitumor property increased against cancer cells *in vitro* and *in vivo*. Thymoquinone is shown to boost the anticancer effect of doxorubicin in certain cancer cell lines [16]. In combination with a single dose of radiation (2.5 Gy), TQ exerted supra-additive cytotoxic effects on breast carcinomas as measured by cell proliferation, colony formation and flow cytometer–based apoptosis assays [17].

In this study we investigate the antitumor activity of thymoquinone (TQ) and/or diosgenin (DG) in A431, Hep2 and RPMI 2650 squamous cell carcinoma (SCC) cells *in vitro* and sarcoma 180–induced solid tumors *in vivo*. We look into the detail of the inhibition effect on proliferation, morphology, cell cycle, DNA fragmentation, activation of caspases, cleavage of poly (ADP) ribose polymerase, Bax and/or Bcl-2, phospho-JNK and -Akt regulations.

## Materials and Methods

### Chemicals and reagents

Dulbecco's modified Eagle medium (DMEM), modified essential medium (MEM), foetal bovine serum (FBS), LIVE/DEAD Viability/Cytotoxicity assay kit (Invitrogen Corporation, Carlsbad, CA USA), *in situ* cell death detection kit (Roche, Mannheim, Germany), Trypan blue, thiazoyl blue tetrazolium bromide (for the MTT assay), thymoquinone (TQ) and diosgenin (DG), propidium iodide (PI), 4′,6-diamidino-2-phenylindole (DAPI), phalloidin, tetramethylrhodamine B isothiocyanate (TRITC), and Annexin V–fluorescein isothiocyanate (FITC)/PI (Sigma-Aldrich, St. Louis, MO, USA), antibodies (Cell Signaling Technology Beverly, MA, USA) and other chemicals were purchased from Sigma-Aldrich, St. Louis, MO, USA; Himedia India, Ltd., Mumbai, India; and Merck India, Ltd., Mumbai, India were purchased for the experimentation.

### Cell culture

Human SCC A431, Hep2 and RPMI 2650 cells were obtained from National Center for Cell Science (Pune, India) and HaCat were obtained from American Type Culture Collection (ATCC) 10801 University Boulevard Manassas, VA 20110, USA. The cells were cultured in DMEM and MEM supplemented with 10% heat-inactivated FBS and 1% penicillin streptomycin (Himedia). The cells were incubated at 37°C in a humidified atmosphere containing 5% CO_2_ and 95% air.

### Cytotoxicity assay

Cells (2.5×10^3^) were seeded in 200 µL medium per well in 96-well plates and were incubated at 37°C in 5% CO_2_ for 24 hours to induce cell adherence. Cells were treated with different concentrations of TQ and/or DG (control cells with vehicle only) and incubated at 37°C in 5% CO_2_ for 48 or 72 hours. Eight wells (n = 8) used in the 96 well plate for each concentration of TQ and/or DG treatment. For the MTT assay, thiazolyl blue tetrazolium bromide solution (100 µL; 1 mg/mL) in incomplete medium was added and this mixture incubated for 6 hours. After this, 100 µL dimethylsulphoxide (DMSO) was added and the plates put on a shaker for 5 minutes. Optical density was recorded at 560 nm with DMSO as the blank [18].

### Morphological analysis by phase-contrast microscopy

A431, Hep2 and HaCat cells at a density of 1.0×10^4^ were grown on sterile poly-L-lysine-coated glass cover slips and treated with different concentrations of TQ and/or DG for 48 hours. After incubation, treated cells and untreated controls were observed under a phase-contrast microscope (Leica, Solms, Germany) [19].

### Cell cycle analysis

Propidium iodide is the most widely used dye for analysis of cell cycle or DNA content. Propidium iodide binds to the major groove of double-stranded DNA and double-stranded RNA, but for DNA it is necessary to treat the cell with RNase for optimal DNA resolution. It produces a highly fluorescent adduct which has an excited wavelength of 488 nm and emission wavelength of 600 nm. Cells (1.25×10^5^) were seeded in 60-mm cell culture dishes and incubated until the cells adhered. After reaching 60–70% confluence, cells were treated with one of both of the drugs for the indicated time intervals and were then harvested by using a trypsin/EDTA mixture. Cells were washed once with phosphate-buffered saline solution (PBS) and fixed with 70% ethanol overnight at −20°C. Finally, cells were stained with PI (1 mg/mL) for 30 minutes and the fluorescence was analyzed immediately by flow cytometry [18].

### Cytoskeletal and nuclear analysis by fluorescence microscopy

Cytoskeleton analysis of A431 and Hep2 cells was performed under a Zeiss Observer Z1 microscope using ApoTome mode (Carl Zeiss, Oberkochen, Germany). Briefly, cells were grown on poly-L-lysine-coated glass cover slips and treated with respective drugs for 48 hours. The medium was removed, and slides were washed three times with PBS (pH 7.4), fixed with 4% paraformaldehyde in PBS (pH 7.4) and permeabilized with 0.1% Triton X-100. Nonspecific binding sites were blocked by applying PBS containing 10% FBS for 1 hour. The cells were stained with DAPI/phalloidin or TRITC to visualize nuclear or cytoskeletal actin, respectively. After staining, the adhering cells were washed with PBS, air dried and mounted on slides. Fluorescent images from the stained constructs were obtained by using ApoTome mode at 20×.

For nuclear analysis, the protocol just described was followed with slight modification. Briefly, cells were treated with respective drug for 48 hours, fixed, permeabilized, stained with 0.2 mg/mL of DAPI in PBS at room temperature for 15 minutes and mounted onto glass slides. The nuclear morphology of the cells was analysed by using an epi-fluorescence microscope at 20× (Leica). Apoptotic cells were identified on the basis of nuclear morphology and chromatin condensation and fragmentation [19].

### DNA fragmentation by agarose gel electrophoresis

Control and treated cells were subjected to lysis in a buffer containing 10 mM Tris (pH 7.4), 150 mM NaCl, 5 mM EDTA and 0.5% Triton X-100 for 45 minutes on ice. Lysates were subjected to vortexing and then centrifugation at 10,000 *g* for 20 minutes. DNA in the supernatant was extracted with an equal volume of a neutral phenol∶chloroform∶isoamyl alcohol mixture (25∶24∶1) and then was examined electrophoretically on 1.8% agarose gels containing 0.1 µg/mL ethidium bromide [20].

### *In situ* cell death detection

For detection of apoptotic cells, a terminal deoxynucleotidyl transferase (TdT)-mediated dUTP nick end labelling (TUNEL) stain was performed using the *In Situ* Cell Death Detection Kit–Fluorescein (Roche Molecular Biochemicals, Chemicon Int., Temecula, CA, USA) according to the manufacturer's instructions. Cells were grown on poly-L-lysine-coated glass cover slips and treated with respective drugs for 48 hours. The medium was removed, and slides were washed three times with PBS (pH 7.4), fixed with 4% paraformaldehyde in PBS (pH 7.4) and permeabilized with 0.1% Triton X-100. Aliquots (50 µL) of the reaction mixtures were applied to cover slides and placed in a humidified incubator at 37°C for 60 minutes. After incubation, cells were washed with PBS, air dried and mounted on slides. The slides were examined by epi-fluorescence microscopy [21].

### Combination index of TQ and DG

The cytotoxic effects obtained with the different concentrations of TQ and/or DG were analysed by the method of Chou and Talalay, 1984 [22] on CalcuSyn software (Biosoft, Cambridge, UK). For combinations of TQ and DG, interactions between the two were assessed by means of an automatically computed combination index (CI). The CI was determined at a given effect (F) for combination of cytotoxic A and cytotoxic B by the following equation:
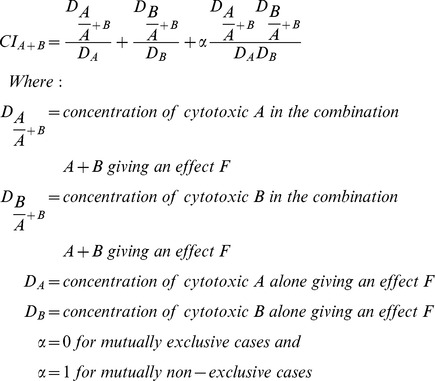
The CI provides a quantitative measure of the degree of drug interaction in terms of additive effect (CI = 1), synergism (CI<1), or antagonism (CI>1) for a given endpoint of the effect measurement [23].

### Immunoblotting analysis

Cells were scraped from the culture, washed twice with PBS and then suspended in 30 µL of immunoblotting lysis buffer containing 50 mM Tris-HCl (pH 7.5), 250 mM NaCl, 5 mM EDTA, 1 mM EGTA, 1 mM NaF, 1 mM phenylmethylsulphonyl fluoride, 1 mM dithiothreitol, 20 µg/mL leupeptin, 20 µg/mL aprotinin, 0.1% Triton X-100 and 1% sodium dodecyl sulphate (SDS) at 0–4°C for 15 minutes. After centrifugation at 15,000 *g* for 10 minutes at 0°C, the supernatants were collected, and the protein amount in each sample was measured by a Bio-Rad DC kit (Bio-Rad, Hercules, CA). Equal amounts of sample (60 µg of protein) were subjected to electrophoresis on either 12% or 15% SDS-polyacrylamide gel, and the gel was transferred to a nitrocellulose membrane, which was blocked by 3% bovine serum albumin in Tris-buffered saline Tween 20 solution and probed with the appropriate antibodies and secondary antibodies. Membranes were then developed using an enhanced chemiluminescence reaction system according to the manufacturer's recommendation [18].

### Treatment in a sarcoma 180–induced mouse model

Swiss albino mice (6–8 weeks old and weighing 18–22 g) were maintained under pathogen-free conditions and standard temperature (25±2°C) and humidity (60±5%) with alternating 12-hour light/dark cycles. The animals were fed a standard pellet diet and maintained in accordance with the guidelines of the National Institute of Nutrition, Indian Council of Medical Research (Hyderabad, India), and in conditions approved by the ethical committee of Indian Institute of Technology (Kharagpur, India). Sarcoma 180 cells were collected at their logarithmic growth phase and suspended in serum-free medium at density of approximately 10^6^ cells in 0.2 mL, the volume of the suspension injected intradermally into the right flank of each mouse. When tumor volumes reached 90–100 mm^3^ (after 20 days), the animals were randomly assigned into four groups (five animals per group). Prior to treatment, body weight and tumor volume of all tumor-bearing mice were measured. Then, the mice were injected by tail vein as follows: (i) sterile isotonic saline solution (control group); (ii) TQ (10 mg/kg/day); (iii) DG (20 mg/kg/day); or (iv) TQ (10 mg/kg/day)+DG (20 mg/kg/day). During the first 9 days injections were performed 6 times, once per day, one day spaced between two administrations; after that, injections were given according to the same schedule but every alternate day up to 18 days [24].

At the end of the treatment period, the mice were asphyxiated by breathing carbon dioxide. Their tumors were excised and measured with vernier calipers in two dimensions. The tumor volume (V in mm^3^) was calculated according to the following formula (18): V = (L×W^2^) π/6, where L (mm) is the longest diameter and W (mm) is perpendicular to L. For immuno-histochemical analysis, one part of the tumor was fixed in formalin and embedded in paraffin. Another part was embedded in optimal cutting temperature compound, rapidly frozen in liquid nitrogen and stored at −80°C.

### Immunohistochemical analysis

Immunohistochemical analysis of cellular proliferation (Ki-67, CD31 antigens) of sarcoma 180–induced tumors in mice was performed using Ki-67 and CD31 antibodies. Tissue specimens were processed for immunohistochemical analyses as described previously. Neutral buffered formalin-fixed tissue was embedded in paraffin. Tissue sections (5 mm) were prepared with a microtome and mounted on slides, and immunohistochemical analysis was done within 24 hours. Sections were deparaffinized in xylene, rehydrated in graded alcohols (100%, 95% and 80% v/v) and washed in distilled water. Endogenous peroxidase activity was quenched with 0.01% H_2_O_2_. Sections for Ki-67 and CD31 analysis were treated further with 0.05% trypsin and 0.05% CaCl_2_ in Tris-HCl (pH 7.6) for 5 minutes at 37°C. Antigen retrieval was done by microwaving the sections in 10 mM/L citric acid (pH 6.0) for 30 minutes. The slides were washed thrice in PBS and blocked with 10% normal horse serum for 30 minutes. Tissue sections were then incubated with antiserum to Ki-67 and CD31 (1∶50) for 3 hours at room temperature. After being washed thrice with PBS, the sections were incubated with appropriate secondary immunoglobulins (1∶500) for 45 minutes at room temperature. The slides were then washed thrice in PBS, labelled with avidin-biotin peroxidase complexes (1∶25) for 30 minutes at room temperature and then washed with 2× PBS. Immunoreactivity was determined using diaminobenzidine as the final chromogen. Finally, sections were counterstained with Meyer's hematoxylin, dehydrated through a sequence of increasing concentrations of alcohol, cleared in xylene and mounted with epoxidic medium [18].

The effects of TQ and DG on apoptosis in the mouse tumors were determined by using a commercially available TUNEL kit (Roche) and DAPI staining. The TUNEL/PI assay was as already described, with the following modifications. Sections from frozen tissues were fixed with 4% paraformaldehyde for 10 minutes, washed twice with PBS for 5 minutes and then incubated with 0.1% Triton X-100 for 15 minutes. After two 2-minute washes with PBS, the samples were incubated with equilibration buffer for 10 minutes. The equilibration buffer was drained; the sections were treated with reaction buffer and incubated at 37°C for 1 hour in the dark. Samples were then washed with PBS to remove unincorporated fluorescein-dUTP. The cells were counterstained with PI. TUNEL-positive apoptotic cells were detected by localized green fluorescence within the nucleus. Some tissue sections were stained with 10 µg/mL DAPI in PBS for 10 minutes at room temperature, washed twice with PBS, counterstained with phalloidin and mounted. Apoptotic cells were identified on DAPI/phalloidin–stained images by morphological changes of the nuclear actin filaments and chromatin condensation and fragmentation [18].

### Statistical analysis

The data were analysed to obtain mean values and standard errors for all treated and vehicle control samples; the means were subjected to statistical comparison using the Student *t-*test (*P*<0.05 was considered significant). Statistical analysis was done with the help of Prism 5.01, GraphPad Software Inc. (La Jolla, CA, USA).

## Results

### Inhibition of growth and proliferation by TQ and DG

**Figure 1:**
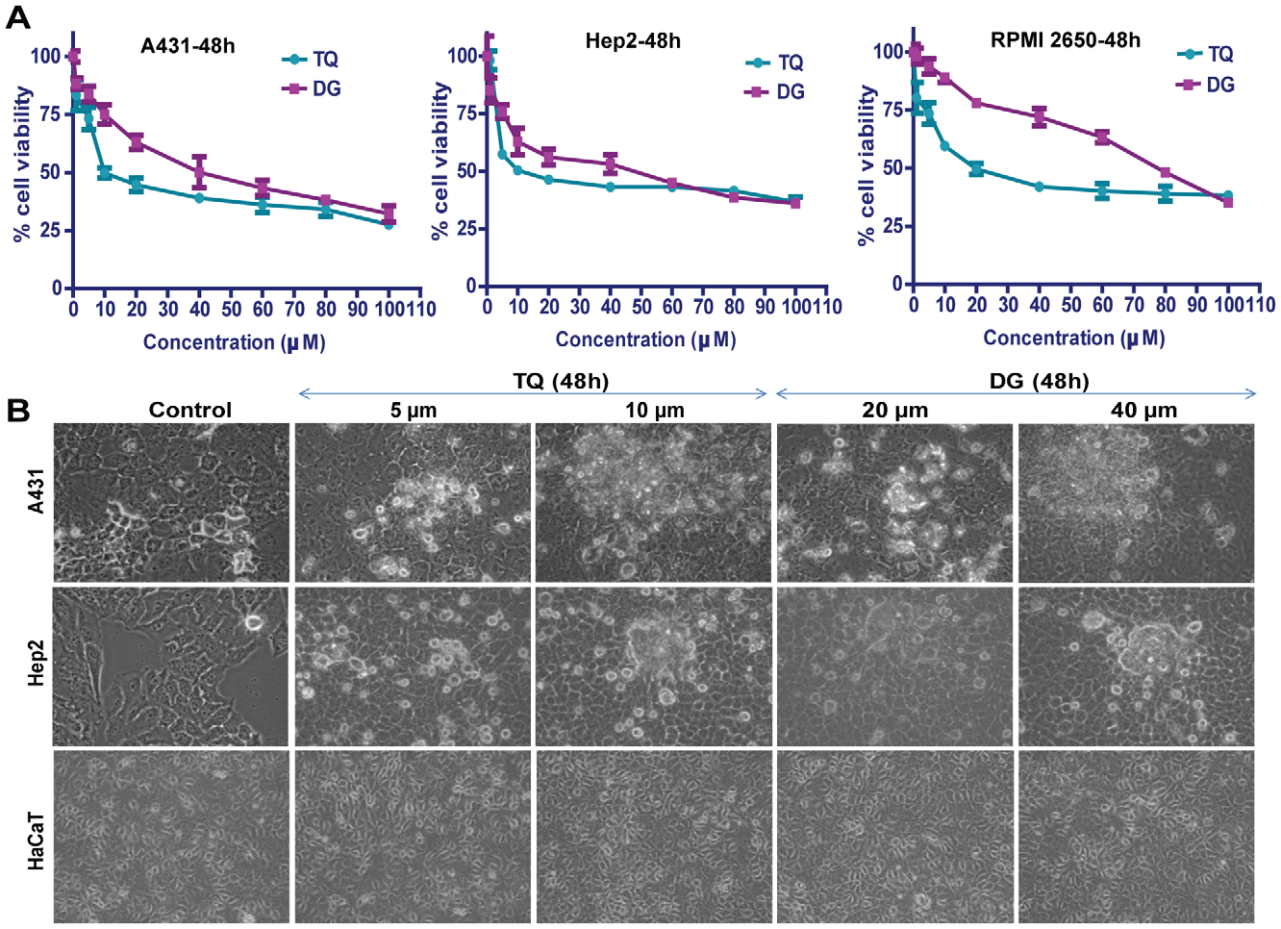
Dose-dependent growth inhibition of squamous SCC cells by thymoquinone (TQ) and diosgenin (DG). (A) A431, Hep2 and RPMI 2650 cells were treated with different concentrations of TQ or DG for 48 hours and MTT assays were performed. Points represent means ± SEM, n = 8 in three different experiments. (B) Phase-contrast photomicrographs (Leica 4×) of A431, Hep2 and HaCaT cells treated with different concentrations of TQ or DG for 48 hours.

The cytotoxic effects of TQ and DG on A431, Hep2 and RPMI 2650 cells were determined by treating cells with varying concentrations of the compounds (2–100 µM) for 48 hours, which revealed dose-dependent inhibition of cell growth and proliferation. The data indicate that treatment of cells with different concentrations of these two agents resulted in significant inhibition of cell viability as compared to controls (Figure 1A). TQ and DG were found to inhibit the proliferation of A431 and Hep2 cells in a dose-dependent manner, with median inhibitory concentrations (IC_50_) of 10 µM and 40 µM, respectively, whereas the IC_50_ for RPMI 2650 were almost double those. On the basis of the proliferation assay, we selected A431 and Hep2 for further experiments.

### Morphological analysis

Phase-contrast microscopic analysis of TQ- and DG-treated A431, Hep2 and HaCaT cells revealed significant morphological changes in A431 and Hep2 cells treated with different concentrations of each drug as compared to respective controls. These include retraction of cellular processes and cell shrinkage. In contrast, the control cells were well spread with a flattened morphology. The human keratinocyte cell line HaCaT showed no morphological alterations in response to drug treatment, which confirms that TQ and DG have no cytotoxicity in normal cells (Figure 1B).

### Flow cytometer–based apoptosis assay

**Figure 2:**
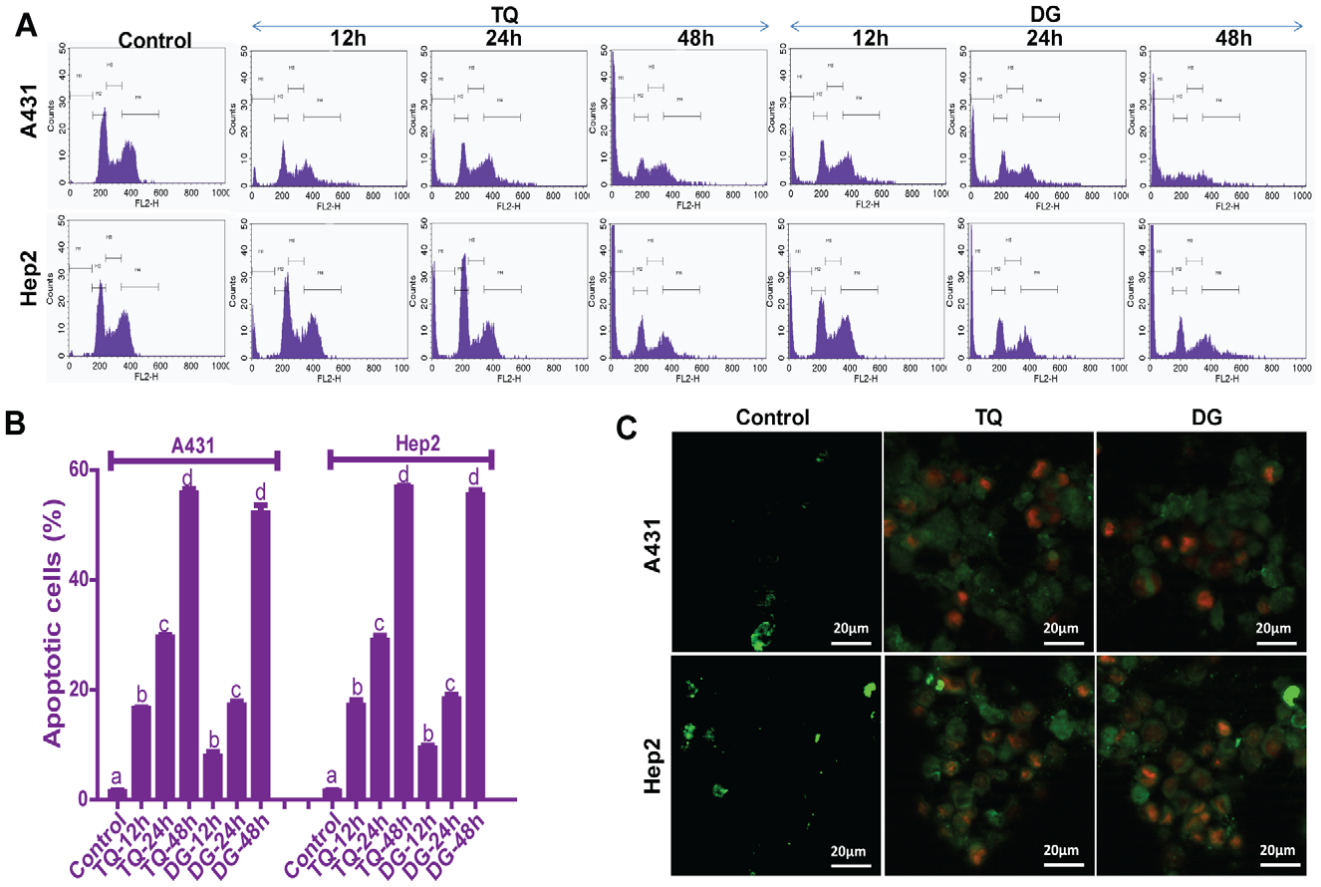
TQ- and DG- induce apoptosis in A431 and Hep2 cells. (A) Dose-dependent effects of TQ and DG detected by cell cycle–based apoptosis assay. Cells were treated with respective IC_50_ of TQ or DG for 48 hours, stained with PI and measured by flow cytometry. (B) Accumulation of sub-G_1_ cells plotted from the cell cycle–based apoptosis assay. Data are presented as means ± SEM from three independent experiments. Dissimilar superscripts (a, b, c, d) represent the significant differences among them (*P*<0.05). (C) After treating with IC_50_ of TQ or DG, cells were stained with Annexin V-FITC and PI and analysed by fluorescence microscopy. Three primary populations of cells: cells are viable and not undergoing apoptosis (Annexin V-FITC and PI negative), cells undergoing apoptosis (Annexin V-FITC positive and PI negative) and cells in end-stage apoptosis or already dead (Annexin V-FITC and PI positive).

DNA content of treated A431 and Hep2 cells was analysed by using PI staining, and cell distributions among sub-G_1_, G_0/1_, S and G_2_/M phases were expressed. Cells were treated with the IC_50_ concentrations of TQ and/or DG for 12, 24 or 48 hours. Results revealed increasing accumulation of cells at the sub-G_1_ phase over time. Accumulation of sub-G1 phase cells was significantly greater in cells treated with either drug than in controls (Figures 2A, B), corroborating the results of the MTT cell proliferation assay. Human normal HaCaT keratinocytes treated with TQ or DG (10 µM and 40 µM, respectively) for 48 hours showed no significant apoptotic population (data not given).

### Epi-fluorescence microscopic analysis of apoptosis

For confirmation of the apoptosis-inducing activities of TQ and DG, cells were stained with Annexin V-FITC and PI and analysed by fluorescence microscopy. A431 and Hep2 cells were left untreated or were treated for 48 hours with TQ or DG at respective IC_50_ doses. Untreated control cells were primarily negative for Annexin V-FITC and PI, indicating that they were viable and not undergoing apoptosis. Among the treated groups were cells that were Annexin V-FITC positive and PI negative, indicating that they were undergoing apoptosis, and cells that were Annexin V-FITC and PI positive, indicating that they were in end-stage apoptosis or already dead (Figure 2C).

### Cytotoxicity assay

**Figure 3:**
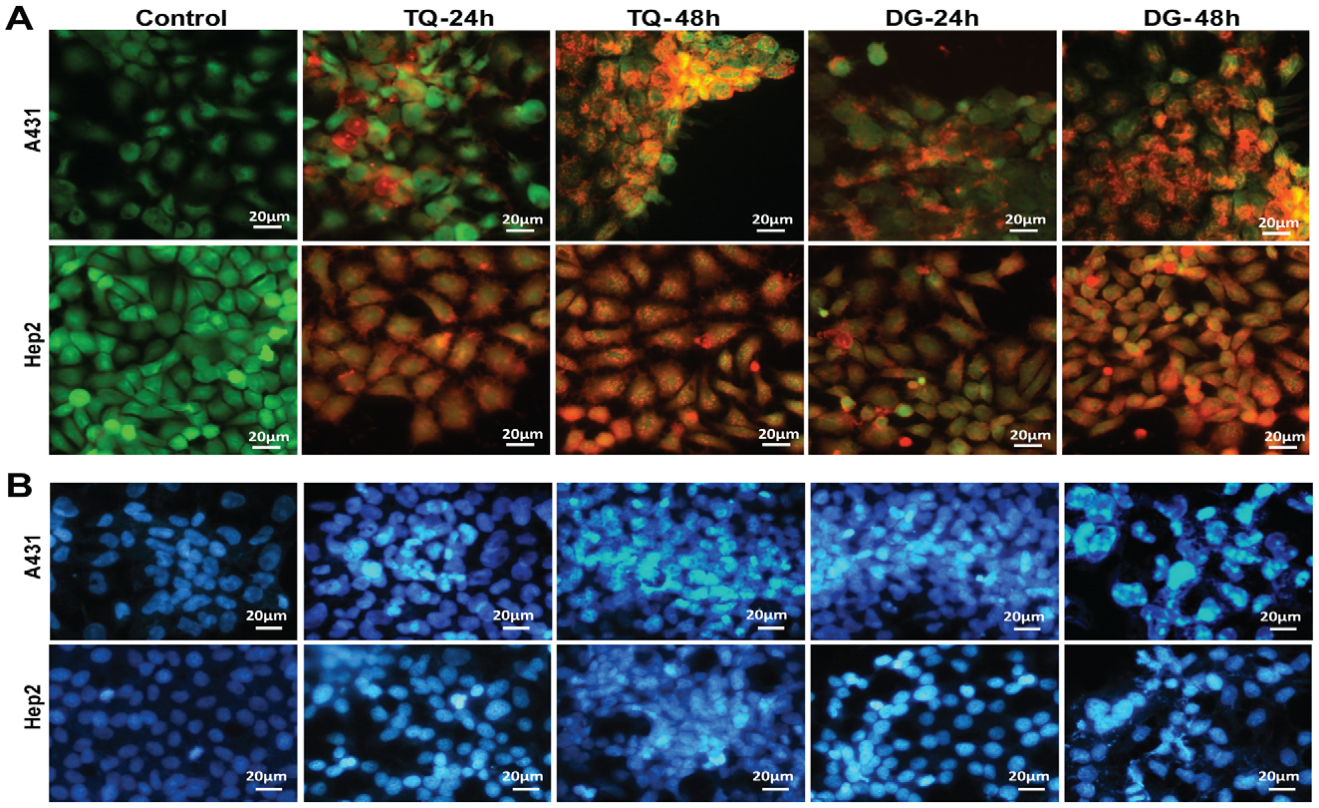
Fluorescence-based cytotoxicity assay and chromatin condensation analysis. (A) Fluorescence-based cytotoxicity assay (using the LIVE/DEAD Viability/Cytotoxicity kit) of A431 and Hep2cells treated with TQ or DG for 24 or 48 hours. A significantly higher percentage of apoptotic cells (live (green)/dead (red)) are observed in the treated groups than in controls. (B) Fluorescence microscopic observation of A431 and Hep2 treated with IC_50_ of TQ and DG for 24 or 48 hours and stained with DAPI to detect chromatin condensation.

Cell viability measures the amount of cells that are living or dead, based on a total cell sample. To define the contributory roles of proliferation and apoptosis in cell viability, cytotoxicity assays were performed using the LIVE/DEAD Viability/Cytotoxicity kit. Decreased cell viability was a consequence of both the growth-inhibitory and apoptotic effects of TQ or DG alone over time. Treated cells had >50% apoptotic cells, corroborating the results of the MTT and flow cytometry–based apoptotic assays (Figure 3A).

### TQ- and DG-induced chromatin condensation

To better understand whether TQ- and DG- induced apoptosis in A431 and Hep2 cells, DAPI staining was done. Epi-fluoroscence micrographs revealed distinct oligonucleosomes after DAPI staining confirming chromatin condensation (Figure 3B).

### DNA fragmentation and TUNEL assay of apoptosis

**Figure 4:**
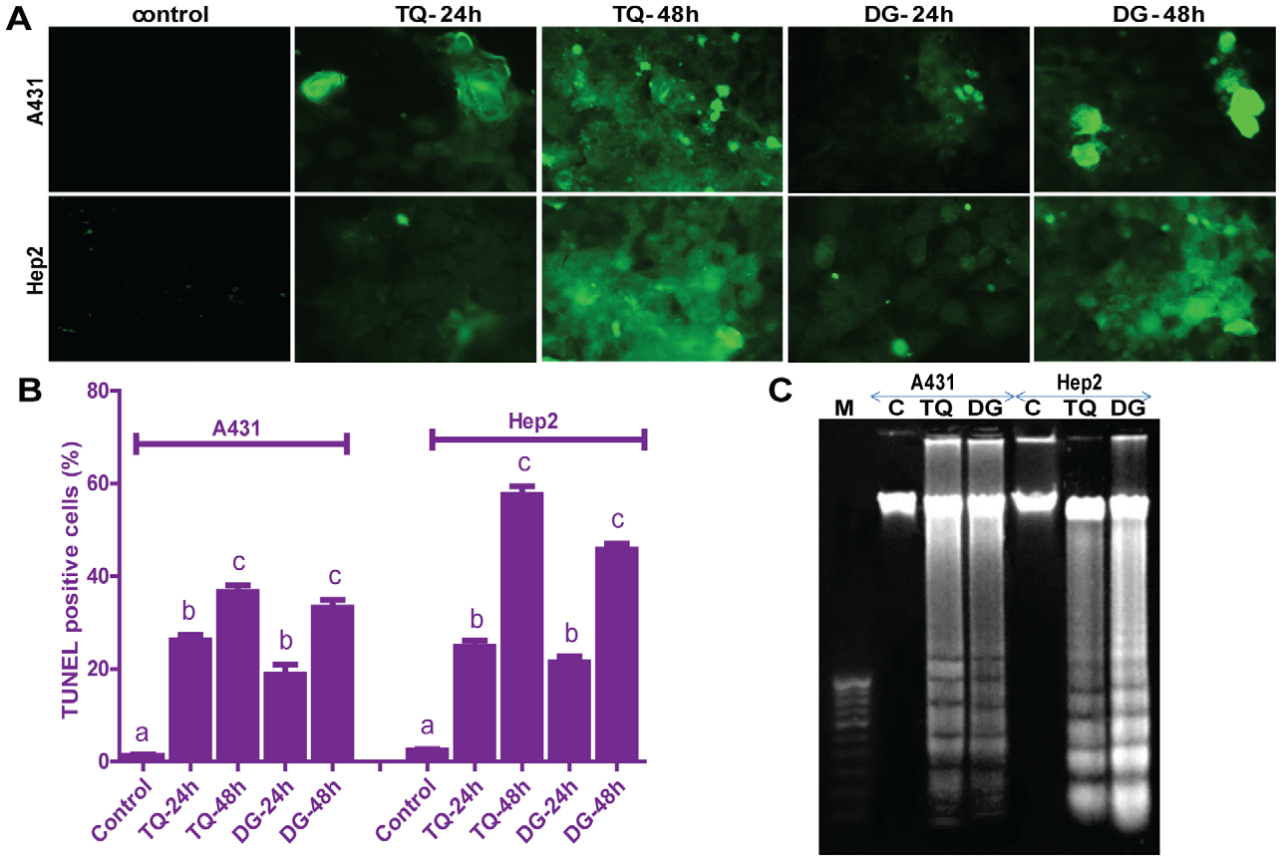
TQ- and DG- induced apoptosis as measured by DNA laddering and TUNEL assay. (A) Representative image of TUNEL assay in TQ- or DG- treated A431 and Hep2 cells. (B) Apoptotic cells are quantified by counting the percentages of TUNEL-positive nuclei. Data are presented as means ± SEM of three independent experiments. Dissimilar superscripts (a, b, c, d) represent the significant differences among them (*P*<0.05). (C) DNA laddering of A431 and Hep2 cells treated with IC_50_ of TQ or DG for 48 hours.

We used TUNEL staining to identify apoptotic cell death induced by TQ or DG. TUNEL-positive nuclei were found throughout the photomicrographs of the treated groups but few in untreated controls, and numbers of positive nuclei increased with treatment time (Figure 4A, B). DNA fragments, which are characteristic of apoptotic cells, were examined in TQ or DG treated A431 and Hep2 cells. DNA laddering patterns were captured for cells untreated or treated with IC_50_ doses of TQ or DG for 48 hours (Figure 4C).

### TQ- and DG-induced alteration of cell-regulatory and apoptotic proteins

**Figure 5:**
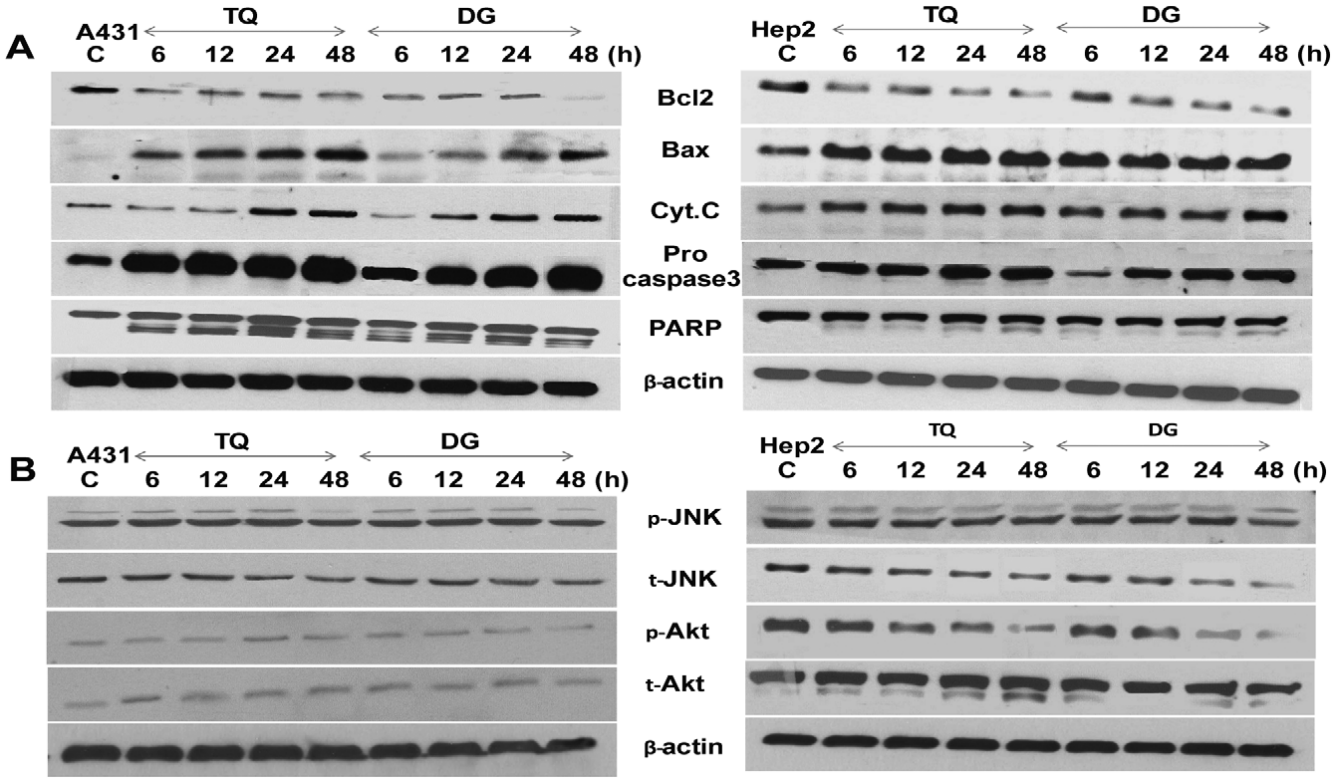
TQ- and DG- induced apoptosis by altering the apoptotic proteins and signalling molecules. (A) Western blot analysis of proapoptotic and antiapoptotic proteins in A431 and Hep2 cells treated with TQ or DG for various durations. (B) Western blot analysis of phosphorylated Akt and JNK during TQ- and DG-induced apoptosis. Beta-actin are used as loading control.

We used immunoblotting to examine the mechanistic pathway of apoptosis in TQ- and DG- treated A431 and Hep2 cells. Following 6, 12, 24 or 48 hours exposure to TQ or DG, cells showed decreases in the inactive 32-kD proform of caspase-3, indicating the role of effector caspase-3 in DNA fragmentation and apoptosis induction. Release of cytochrome *C* and cleavage of PARP were also observed over time. Moreover, the proapoptotic Bax protein was upregulated and the antiapoptotic Bcl-2 protein downregulated in A431 and Hep2 cells in a treatment time–dependent manner. Overall, the Bax/Bcl-2 ratio was increased by treatment, suggesting that this is one of the mechanisms of TQ- and DG-induced apoptosis (Figure 5A).

Cells were treated with TQ or DG for different time intervals and then were subjected to western blot analysis with antibodies to phospho-JNK, phospho-AKT (Ser^473^), total JNK and total AKT. Levels of phospho-JNK and phospho-AKT were markedly reduced by treatment with TQ or DG in a time-dependent manner in both A431 and Hep2, whereas total levels of JNK and AKT remained unaltered by treatment with TQ or DG (Figure 5B).

### TQ synergism with DG and vice versa

**Figure 6:**
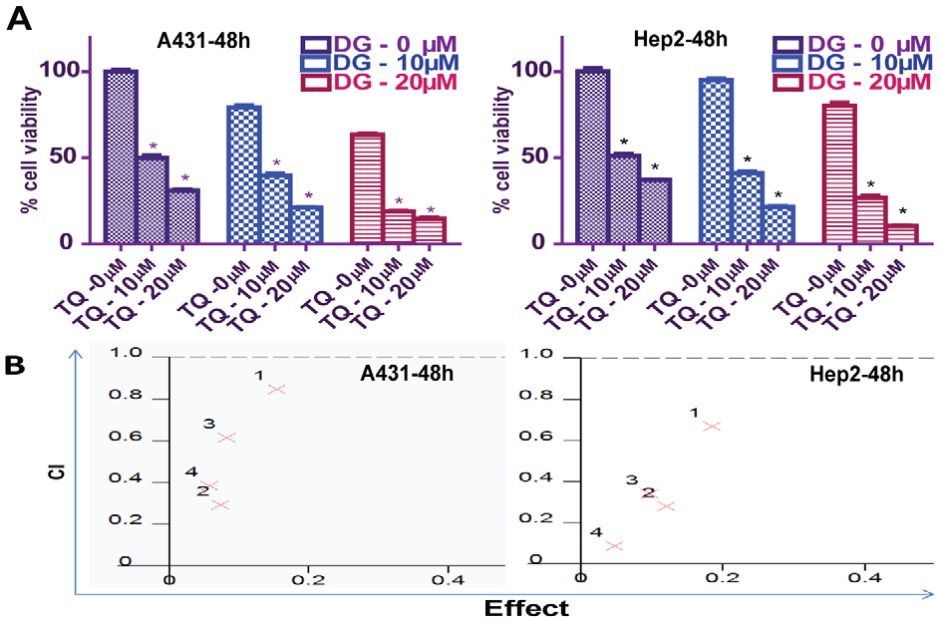
Dose effects on cell proliferation in studies of TQ and DG combinations. (A) MTT assay of proliferation of cells treated with different concentrations of TQ and DG for 48 hours. Points represent means ± SEM of three different experiments. (B) Combination index (CI) was calculated; CI<1 suggests synergism between TQ and DG in A431 and Hep2 cells. Red crosses (1–4) represent the concentrations of TQ and DG in various combinations: 1 = 10 µM∶10 µM (TQ∶DG); 2 = 10 µM∶20 µM (TQ∶DG); 3 = 20 µM∶10 µM (TQ∶DG); and 4 = 20 µM∶20 µM (TQ∶DG).

**Figure 7:**
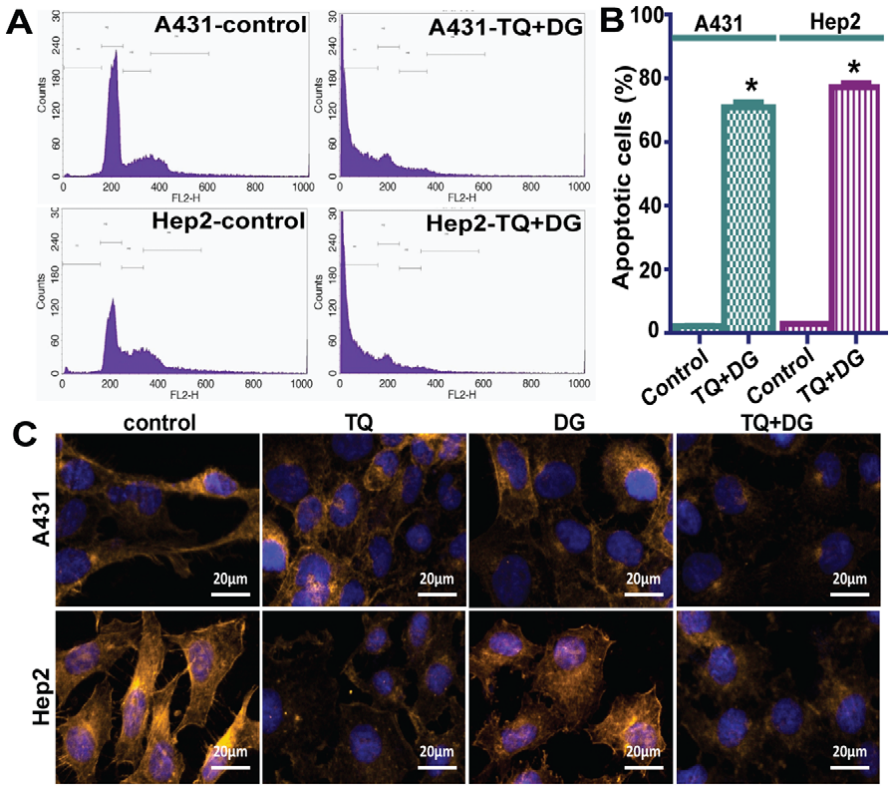
Combination of TQ and DG enhances these agents' antiproliferative and apoptotic effects in SCC cells. (A) Flow cytometry–based detection of apoptotic cell death in A431 and Hep2 cells treated with a combination of TQ 10 µM and DG 20 µM. (B) Accumulation of sub-G_1_ phase apoptotic cells during treatment with the same combination of TQ 10 µM and DG 20 µM. Data represent means ± SEM; *represents significant differences between control and treated groups (*P*<0.05). (C) A431 and Hep2 cells treated with TQ and/or DG for 48 hours, labelled with phalloidin-TRITC (F-actin–binding protein, golden) and DAPI (DNA-binding dye, blue). TQ or DG alone effectively blocked cell-spreading lamellopodia, whereas combination treatment led to complete disorganization of actin filaments and formed F-actin fragments.

In the cell proliferation assay, TQ or DG alone (10 and 40 µM, respectively) for 48 hours significantly inhibited cell proliferation. DG alone at doses of 10 and 20 µM for 48 hours also inhibited proliferation of A431 (79.21% and 63.47% viable, respectively) and Hep2 (95.01% and 80.27% viable, respectively), suggesting that more frequent dosing of DG may be required to demonstrate a sustained effect. The combined effect of TQ and DG on cell proliferation was most noticeable at respective doses of 10 and 20 µM. In A431 and Hep2 cells, treatment with the combination of TQ 10 µM and DG 20 µM reduced cell viability remarkably, to 18.87% and 26.61%, respectively, significantly lower than in controls. The combination of TQ 20 µM and DG10 µM reduced A431 and Hep2 viability to 21.19% and 21.85%, respectively, again significantly lower than in controls. The combination of TQ 20 µM and DG 20 µM at 48 hours reduced viability to 14.71% and 10.38% in A431 and Hep2 cells, respectively (Figure 6A). That the decreased cell viability was a consequence of both the growth-inhibitory and combined apoptotic effects of TQ (10 µM) and DG (20 µM) was confirmed by flow cytometry (Figure 7A, B).

When the A431 and Hep2 cells were grown in the presence or absence of TQ and/or DG, it was apparent that the combined effect of TQ and DG was greater than the effect of each agent alone. To confirm the presence of synergism, we determined the CI for two combination treatment groups in both A431 and Hep2 cells; CI<1 indicated a synergistic effect (Figure 6B). Synergism was detectable in all four combinations treated for 48 hours (Table 1).

**Table 1:** Synergistic effect of TQ and DG in squamous cell carcinoma cells.

Concentration (µM)		CI	
TQ	DG	A431	Hep2
10	10	0.847	0.67
10	20	0.293	0.282
20	10	0.615	0.342
20	20	0.386	0.088

Combination index (CI) values are calculated according to the Chou and Talalay (1984) mathematical model for drug interactions using the Calcusyn software.

### Cytoskeletal structure during combination treatment with TQ and DG

The cytoskeletal structure was analysed with phalloidin-TRITC and DAPI staining in A431 and Hep2 cells during treatment with TQ and/or DG. We attempted to elucidate the cytoskeletal remodeling and generation of membrane protrusions, such as pseudopodium, filopodia and ruffle formations, by fluorescence microscopy (ApoTome mode) in A431 and Hep2 cells treated with TQ and/or DG. The control cells displayed elongated shapes and dense networks of actin forming organized, parallel filamentous structures.

Long, stressed F-actin filaments, round in shape, sparse and irregular with no striations, were observed in A431 and Hep2 cells treated with TQ or DG. On the other hand, cells treated with the combination of TQ and DG revealed highly irregular actin filament organization and loss of filopodia and lamellipodia. The loss or intense collapse of cytoskeleton organization in A431 and Hep2 cell lines treated with the combination proved the potential for apoptosis (Figure 7C).

### Tumor growth in mice bearing sarcoma 180–induced solid tumor

**Figure 8:**
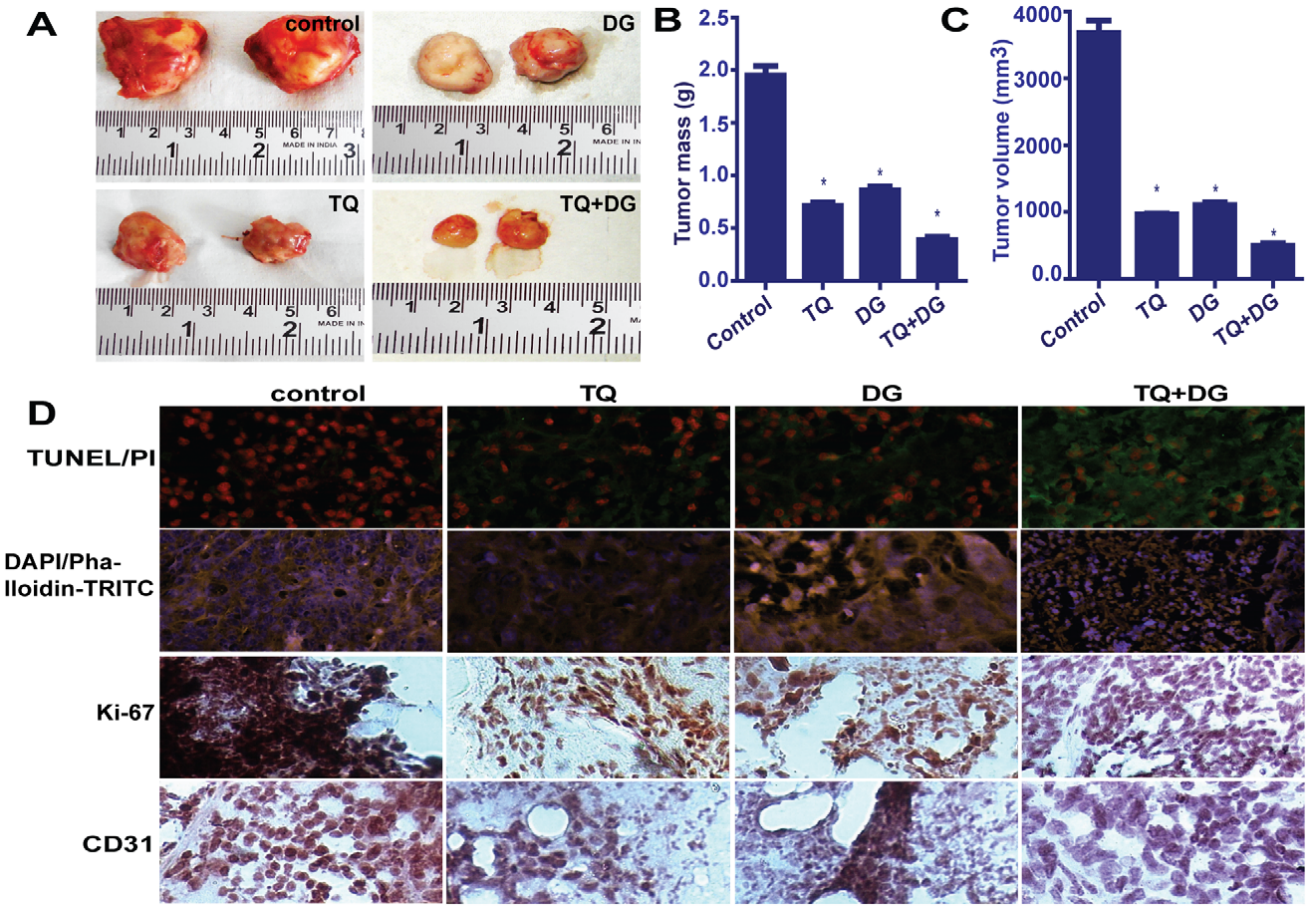
Antineoplastic activities of TQ and/or DG in sarcoma 180-induced xenografts in a mouse model. Mice bearing sarcoma 180 xenografts are given tail vein injections of TQ (10 mg/kg/day) and/or DG (20 mg/kg/day) for up to 27 days. (A) Before treatment, tumors are prominent and large in both controls and treated groups. After treatment, mice are killed and their tumors are excised, measured and weighed. (B) Bar graph represents tumor masses in grams. (C) Data points represent tumor volumes for control and treatment groups. Data represent means ± SEM; * represents significant differences (*P*<0.05, n = 5) between control and treated groups. (D) Immunohistochemical analysis of sarcoma 180–induced tumor xenografts from mice treated with TQ and/or DG. Paraffin-embedded sections of tumors are processed and assays of TUNEL/PI and DAPI/phalloidin-TRITC and immunohistochemical assays of Ki-67 and CD-31 are performed.

The effect of TQ and/or DG on solid tumors induced by sarcoma 180 tumor cells in Swiss albino mice was tested, and the results are shown in Figure 8. Treatment with TQ and/or DG significantly reduced tumor size and tumor mass (*P*<0.05) compared to controls (Figure 8A, B). Reductions in tumor volume are summarized in Figure 8C. Immunohistochemical staining of the sectioned tumor masses showed decreased expression of CD31 in TQ- and DG-injected tumors, which suggests the antiangiogenic effects of these drugs. Immunohistochemical analysis of Ki-67 confirmed the antiproliferative functions of TQ and DG, and TUNEL/PI assays confirmed the combined apoptosis-promoting effects of these two agents (Figure 8D).

## Discussion

Natural agents' thymoquinone (TQ) and diosgenin (DG) are proven to inhibit the growth of many different types of tumor cells [4]–[7], [13], [25]. However, their antineoplastic effects in squamous cell carcinoma (SCC) are not well documented. We demonstrate here that TQ and DG have antiproliferative and apoptotic effects on SCC cell lines A431, Hep2 and RPMI 2650, and that these effects are both dose and time dependent. We also observe that TQ and DG potentiate the anticancer effect of each other synergistically *in vitro* and *in vivo*. The ability of TQ to cause synergistic cytotoxicity in combination with chemotherapeutic agents are reported in other cell lines, including BGC-823, MCF7, KBM-5, HL-60 and NCI-H460 [4]–[5], [16], [26]–[27]. Our findings suggest that TQ and DG have potential as complementary natural chemotherapeutic drugs, enhancing the antitumor effects of conventional chemotherapeutic agents. Moreover, TQ shows to ameliorate cisplatin-induced nephrotoxicity and doxorubicin-induced cardiotoxicity in animal models, possibly by acting as a superoxide radical scavenger that inhibits lipid peroxidation [28], [29].

*In vitro* data indicate dose- and time-dependent inhibition of proliferation of human SCC A431 and Hep2 cells by TQ and DG. DG exhibits cytotoxicity at higher concentrations (40 µM) than TQ (10 µM). Cell cycle checkpoints and apoptosis play key roles in developmental biology and represent a new set of potential targets for chemotherapeutic agents. Cell cycle analyses of the TQ- and DG-treated SCC cells show significant treatment time–dependent arrest at the sub-G_1_ phase. These findings establish the antiproliferative and apoptosis-inducing abilities of TQ and DG in A431 and Hep2 cells (Figures 1, 2). Time-dependent TQ-induced sub-G_1_ accumulation is also observed in MCF7 and HL60 cells [5], [30]. Photomicrographs of A431 and Hep2 cells subjected to the LIVE/DEAD viability assay after treatment with TQ and DG further demonstrate the antiproliferative effects of these agents (Figure 3).

Apoptosis induction by TQ and DG is verified by DNA ladder and TUNEL assays. TQ- and DG-treated cells show characteristic ladder-like DNA patterns, unlike respective untreated control cells. Similarly, TQ- induced DNA fragmentation in HL60 cells [30]. TUNEL assay is performed to validate the time-dependent TQ- and DG- induced apoptosis. The number of TUNEL-positive cells provided evidence for DNA fragmentation in both A431 and Hep2 cells after 24 and 48 hours. Nuclear fragmentation proved by DAPI, DNA ladder and TUNEL assays confirmed that cell death is due to activation of apoptotic pathways (Figure 4).

Proteins of the Bcl-2 family play a key role in controlling activation of caspases. Data show that Bcl-2 protein is down regulated and Bax up regulated after TQ or DG treatment (i.e., increased Bax/Bcl-2 ratio). This phenomenon might be one of the mechanisms of TQ- and DG-induced apoptosis in A431 and Hep2 cells. TQ-induced increases in Bax/Bcl-2 ratio were also reported in other cancer cells, including MCF7, HL-60 and HCT116 cells [4]–[5]. This increased Bax/Bcl-2 ratio exerts apoptotic stimuli through stimulation of mitochondrial cytochrome *C* release, leading to TQ- and DG-induced activation of caspase-3 and PARP cleavage. Similar observations are reported in HL60 cells undergoing TQ-induced apoptosis [30]. Caspase-3 is partially or totally responsible for cleavage of many key proteins, including the nuclear enzyme PARP. In the current study, we observe a significant time-dependent increase in caspase-3 activity in TQ and DG treated cells. PARP cleavage has been shown to occur early in the apoptotic response as a result of caspase-3 activity. PARP cleavage correlates well with chromatin condensation, which is associated with condensed chromatin in apoptotic cells and precedes detection of actual DNA fragmentation. Here, we observe in treated cells significant increases of cleaved PARP accompanied by chromatin condensation and DNA fragmentation, the hallmarks of apoptosis (Figure 5).

On the other hand, SCC cell lines show high levels of Akt and JNK phosphorylation, indicating that the Akt and JNK signalling pathway play a role in skin cancer progression. The MAPK and PI3K/Akt pathways play important roles in tumor development and progression. Time-dependent down regulation of pAkt expression in treated A431 and Hep2 cells suggests that TQ and DG inhibit Akt-mediated survival signalling in these cells. Similar effects are observed earlier [31], indicating that DG inhibits Akt in other type of cancers [32]. JNK signalling is shown to be involved in cell cycle control and induction of apoptosis. Our results demonstrate that treatment with TQ or DG inhibited JNK phosphorylation significantly, suggesting that the signalling pathways mediated by JNK are suppressed by TQ and DG. Inhibition of the MAPK and PI3K/Akt pathway may have the potential to prevent angiogenesis, proliferation, invasion and metastasis of a wide range of tumors [33].

These findings prove that TQ and DG can inhibit proliferation in A431 and Hep2 cells and induce apoptosis through DNA fragmentation, activation of caspases, cleavage of poly (ADP) ribose polymerase (PARP) and increasing the Bax/Bcl-2 ratio. Moreover, down regulation of phospho-JNK and -Akt were noted in SCC cell lines treated with TQ or DG.

The combinations of TQ and DG tested (1∶1, 1∶2, 2∶1) are also unique in the literature, and the results show a synergistic effect (CI<1) in all cases (Table 1). Similar results are observed in a cell cycle-based analysis of apoptosis (i.e., sub-G_1_ populations). The mode of action of both drugs and of their combinations is mainly apoptotic in nature. The combination therapy also effectively block cell spreading lamellopodia and completely disorganize actin filament as compared to cells treated with TQ or DG alone (Figures 6, 7); these cytoskeletal changes are due to the increased apoptosis [34].

The present study reports the antitumor activity of TQ and/or DG on mice transplanted with sarcoma 180, a mouse-originated tumor and one of the most frequently used cell lines in antitumor research *in vivo* [35]–[36]. We are reporting for the first time the *in vivo* antitumor effects of TQ, DG, or a combination of the two in squamous cell carcinoma. The antitumor effects are determined by measuring the tumor volume and mass, TUNEL/PI and DAPI/phalloidin-TRITC staining and expression of Ki-67 and CD31 in the tumor tissue. We find that TQ+DG show highly significant potential to reduce tumor size and weight in tumor-bearing animals, much higher than either treatment alone or no treatment. Significant increases in DNA fragmentation and disorganization of F-action filaments are shown by TUNEL assay and DAPI/phalloidin-TRITC staining, respectively, in TQ-, DG- and TQ+DG-treated mice compared to control tumor-bearing mice.

Immunohistochemical staining of tumor cells for proliferation-associated proteins provides information about the tumor proliferation rate. The results reveal significant reductions in Ki-67– and CD31-positive cells as compared to control animals. Since CD31 is highly expressed on tumor tissues in a general state of angiogenesis [37]. This suggests that TQ and DG have antiangiogenesis properties (Figure 8). The antitumor activity of TQ with DG is associated with reductions in tumor mass, tumor volume and tumor proliferation rate, all of which are compatible with its antiproliferative and antiangiogenic effects. The findings confirm a role for TQ and DG as antiproliferative, antiangiogenic and apoptosis-promoting agents in *in vivo* solid tumors.

## Conclusions

The results demonstrate that thymoquinone and/or diosgenin have antiproliferative and apoptotic properties mediated through the caspase, JNK and Akt (Ser^473^) pathway in A431 and Hep2 squamous cell carcinoma cell lines. Combinations of the two drugs have synergistic effects and show potential in suppressing sarcoma 180–induced solid tumors. The findings may offer an alternative strategy for development of potential antineoplastic therapies against squamous cell carcinoma.
